# Stand Diameter Distribution Modelling and Prediction Based on Richards Function

**DOI:** 10.1371/journal.pone.0062605

**Published:** 2013-04-30

**Authors:** Ai-guo Duan, Jian-guo Zhang, Xiong-qing Zhang, Cai-yun He

**Affiliations:** 1 State Key Laboratory of Tree Genetics and Breeding, Research Institute of Forestry, Chinese Academy of Forestry, Beijing, China; 2 Key Laboratory of Tree Breeding and Cultivation of State Forestry Administration, Research Institute of Forestry, Chinese Academy of Forestry, Beijing, China; University of East Piedmont, Italy

## Abstract

The objective of this study was to introduce application of the Richards equation on modelling and prediction of stand diameter distribution. The long-term repeated measurement data sets, consisted of 309 diameter frequency distributions from *Chinese fir* (*Cunninghamia lanceolata*) plantations in the southern China, were used. Also, 150 stands were used as fitting data, the other 159 stands were used for testing. Nonlinear regression method (NRM) or maximum likelihood estimates method (MLEM) were applied to estimate the parameters of models, and the parameter prediction method (PPM) and parameter recovery method (PRM) were used to predict the diameter distributions of unknown stands. Four main conclusions were obtained: (1) *R* distribution presented a more accurate simulation than three-parametric Weibull function; (2) the parameters *p*, *q* and *r* of *R* distribution proved to be its scale, location and shape parameters, and have a deep relationship with stand characteristics, which means the parameters of *R* distribution have good theoretical interpretation; (3) the ordinate of inflection point of *R* distribution has significant relativity with its skewness and kurtosis, and the fitted main distribution range for the cumulative diameter distribution of *Chinese fir* plantations was 0.4∼0.6; (4) the goodness-of-fit test showed diameter distributions of unknown stands can be well estimated by applying *R* distribution based on PRM or the combination of PPM and PRM under the condition that only quadratic mean DBH or plus stand age are known, and the non-rejection rates were near 80%, which are higher than the 72.33% non-rejection rate of three-parametric Weibull function based on the combination of PPM and PRM.

## Introduction

Diameter distributions are well known and widely used for describing forest stand diameter structure [1], [2]. Accurate quantification of tree characteristics permits study of the interaction among physical and physiological processes and growth. Quantification of diameter distributions over time allows the manager to relate the parameters of the distribution to stand age or stand density [3]. The stand volume characteristics are calculated using diameter distribution and tree height and volume models [4]. Growth and yield prediction based on the diameter distribution approach has also been widely used [5], [6].

Various probability density functions (PDF) such as normal, log-normal, gamma, beta, Johnson’s S_B_, and Weibull have been widely used to describe the diameter frequency distributions of forest stands over the last 30 yr [4], [7]–[12]. Additionally, in studies of cumulative diameter distribution, different theoretical growth equations such as Logistic, Gompertz, Mitscherlich, Bertalanffy, Schumacher, Korf, Weibull and Richards have been utilized to characterize the diameter structure of forest stands [13]–[19]. Of these, Richards and Weibull equations showed more flexibility than the others, and were respectively the first and the second most accurate [20].

The methods are used to describe diameter distribution can be classified into parametric and nonparametric methods. The abovementioned functions and equations all belong to parametric methods. Nonparametric methods, like percentile prediction method [21], [22] and *k*-nearest neighbor estimation method [23], [24], do not rely on any predefined functional form and adapt to description of multimodal distributions. The disadvantage of nonparametric methods is that their high amount of required reference material is difficult to acquire and time-consuming [25].

Doubtlessly, in parametric methods, the Weibull is the most commonly used probability density function for fitting tree diameter distributions. Since three- parametric Weibull function had been derived by Weibull [26], due to the relative simplicity of expression formula and its flexibility in fitting a variety of shapes and degrees of skew, this function has proved to be a good distribution model. For the estimation of Weibull parameters, many different methods have been applied, such as moment method, maximum likelihood method, percentiles method and nonlinear regression method [5], [11], [27], [28]. With the presentation of advanced analysis software, nonlinear regression method shows its superiority. The best advantage of this method is that parameters can simultaneously accurately be estimated. Additionally, we should acknowledge the facts that iterated function of the three-parametric Weibull distribution is not easy to converge while using Weibull to fit the diameter distribution data, and the correlativity between the parameters estimates and the whole stand characteristics become weak [29], [30]. Thus we wish to explore a new diameter distribution model that overcomes the disadvantages of Weibull and has the advantages of Weibull such as theoretical meanings of parameters and high simulation precision.

The objectives of this study were to explore the application of Richards equation on modelling and prediction of stand diameter distribution, test the theoretical meanings of its parameters, and compare the properties of modelling and prediction for stands diameter distribution between Richards equation and three-parametric Weibull equation using the long-term repeated measurement data sets from *Chinese fir* (*Cunninghamia lanceolata*) plantations in southern China.

## Materials and Methods

### Data Source

*Chinese fir* (*Cunninghamia lanceolata*) stands located in Fenyi city, Jiangxi Province, China, experience a subtropical climate. The longitude is 114°33′E, latitude 27°34′N. Mean annual temperature, rainfall and evaporation are 16.8°C, 1656 mm, and 1503 mm, respectively. *Chinese fir* stands mentioned as follows in the location all are built and authorized by Research Institute of Forestry of Chinese Academy of Forestry and the data originated from our continuous survey. So no specific permits were required for the described field studies, and the field studies did not involve endangered or protected species.

The unthinned stands of *Chinese fir* were established in 1981. Planting density was limited within an optimum range according to managerial purposes. The series of stand planting densities was 1667, 3333, 5000, 6667, 10000 stems ha^−1^. Every planting density had 3 designed replications. Each plot area was 0.06 ha and two adjacent plots were separated by buffer zone. All trees in each plot were marked for continuous measurement. Stem diameter at breast height (Dbh) was measured after tree height reached 1.3 m. All stands were measured every year before reaching 10 years old, and every two years after reaching 10 years old; all stands were measured 10 times. Self-thinning occurred in all stands during the experimental period. The basic information of 150 unthinned stands is described in Table 1. The database obtained from these unthinned stands was used to build models of diameter distribution.

**Table 1:** Description of the data used for model development.

Planting density(stems/ha)	Stands density (stems/ha)	Numbers of stands	Age (year)	Site index (m)^a^	Dbh (cm)	Height (m)
1667	1633∼1667	30	6∼20	12.52∼16.42	7.90∼18.35	5.50∼15.50
3333	3200∼3333	30	6∼20	14.52∼16.92	6.59∼14.07	5.10∼15.2
5000	4267∼5000	30	6∼20	14.07∼14.47	5.59∼12.27	4.65∼13.70
6667	5450∼6667	30	6∼20	12.88∼13.25	5.16∼10.89	4.60∼12.60
10000	5783∼10000	30	6∼20	13.85∼14.23	4.97∼10.75	4.40∼13.20

a The value of site index is equal to the average dominant height of actual stand of Chinese fir plantation at the reference age of 20. Dbh means diameter at breast height.

Another database comprised of 159 diameter frequency distributions was used to test the models. The data came from a thinning study of *Chinese fir* plantations established in the same environment as the above-mentioned unthinned stands. Among the 159 diameter distributions, 63 distributions came from unthinned stands, the remaining came from thinned stands. Because of thinning only being viewed as a management measure that influences stands diameter distribution as same as density, the thinned stands were included in the test database. All thinning was from below. Each plot area was 0.05 ha. The basic information of used stands data for model evaluation is described in Table 2.

**Table 2:** Description of the data used for model evaluation.

Plots	Stands density (stems/ha)	Numbers of stands	Age (year)	Site index (m)^a^	Dbh (cm)	Height (m)
Un-thinned stands	1680∼4800	63	9∼20	13.98∼17.85	6.40∼16.90	5.10∼15.80
Thinned plot	1000∼5380	96	9∼27	13.35∼18.93	5.30∼20.00	4.40∼17.20

a The value of site index is equal to the average dominant height of actual stand of Chinese fir plantation at the reference age of 20. Dbh means diameter at breast height.

### Computation of Observed Cumulative Diameter Distribution

The diameter classes applied were 2 cm wide. Diameter class, k, is defined in absolute scale (e.g., 1–2.9 cm for *k* = 2, 3–4.9 for *k* = 4, etc.), namely, diameter class *k* is the midpoint value of the absolute scale. The frequency of stems in diameter class *k* at stand *i* is given by:
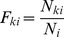



where 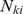 is the number of trees of diameter class *k* at stand *i* (*i* = 1, 2, …, 159), and 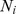 is the total number of trees in stand *i*. The cumulative frequency of stems in diameter class *k* at stand *i* can be obtained by:
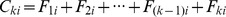



### Model Development

Table 3 shows the basic mathematical characteristics of Richards and Weibull equations. In the expression of Weibull equations, *a* is the location parameter, *b* is the scale parameter, and *c* is the shape parameter. What, then, has caused the iterated function to not easily converge while employing SAS’s nonlinear regression method [31] to estimate the parameters of Weibull and, subsequently, for the correlativity between the parameter estimates and the whole stand characteristics to be weak? When the expression formula of the Weibull equation is analyzed, it is found the equation is meaningful only when
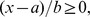



**Table 3:** The basic mathematical characteristics of Richards and Weibull equations.

Equations	Expression formula	Abscissa ofinflection point	Ordinate ofinflection point	Parameter range
Richards (Prototype)	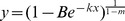	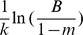	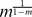	*k,m* >0
*R* distribution	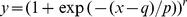	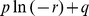	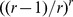	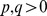 , 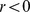
***Weibull***	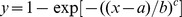	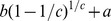	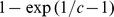	*b,c* >0

because there is b>0, so 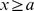, this means the value of parameter *a* should be smaller than the midpoint of minimum diameter class. If 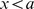, Weibull equation is meaningless. Furthermore, it is difficult to give a suitable initial estimation for the location parameter *a* [32], and the ultimate estimation of the location parameter cannot exceed the two neighboring diameter class of any given initial estimation, which can greatly increase the times of iteration. Obviously, it is the location of shape parameter which leads to the above-mentioned shortcomings of Weibull.

Richards equation have been found to be suitable to fitting the sigmoid data sets [15]. In the beginning, the curve is concave up, while in later life it becomes convex [33], [34]. Therefore, on the basis of prior study, further analysis is needed to determine the mathematical characteristics of Richards. While fitting distribution data, the parameter *B* of Richards is <0. As a result, the following new expression formula of Richards function in Table 3 can be derived:
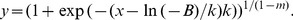
(1)

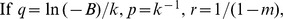
 becomes
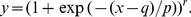
(2)While fitting diameter distributions [15], the empirical values of parameter *B* in Richards function are<−3, then 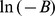
>0, besides, parameter *k* is >0, it can be concluded that parameter *q* is >0, and parameter *p* also is >0. Because parameter *m* is >1, parameter *r* is <0.

Undoubtedly, Eq. (2) will still have high simulation precision, and it is obvious and important that the parameters of Eq. (2) may have the same theoretical meaning as three- parametric Weibull equation. Namely, parameter *q* may be the location parameter, parameter *p* may be the scale parameter, and parameter *r* may be the shape parameter. Note, whether the parameter *q* of Eq. (2) is>*x* or not, problems that the equation has no meaning or parameters have difficulty to converge will not appear. We call Eq. (2) “*R* distribution”. The probability density function of *R* distribution for a random variable *x* is
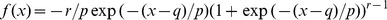
(3)


Eqs. (2) and (3) have the advantage of a simple expression formula. Like Weibull equation, Eq. (2) has a floating inflection point, the abscissa and ordinate of inflection point of Eq. (2) are respectively given by
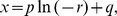


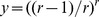



Accordingly, *R* distribution has a good application prospect in the field of diameter distribution. Our database, including 150 cumulative diameter distributions, was used to fit the *R* distribution and Weibull equation, then the theoretical meaning of parameters of *R* distribution was analyzed by discussing the correlativity between the parameters estimates and the whole stand characteristics.

The skewness × kurtosis can describle the shape and modeling properties of distribution function while using for forest stand [12]. The shape of *R* distribution is herein evaluated in terms of its skewness × kurtosis from one side. Skewness and kurtosis values of frequency distributions of original data and *R* distribution are calculated for every 150 stands. The mathematical formulas are:
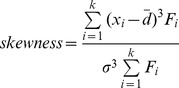


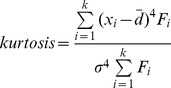



where 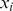 is the midpoint value of diameter class *k*, and 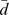 is the average value of DBH of a stand, 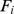 is the frequency of diameter class *k*, 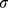 is standard deviation of DBH.

### Model Evaluation

Model evaluation was made through three steps: (1) the parameters of *R* distribution and three- parametric Weibull equstion were estimated by using nonlinear regression method (NRM) or maximum likelihood estimates method (MLEM) for each of the 150 unthinned stands, (2) prediction functions between parameters and stand characteristics were built by using parameter prediction method (PPM) and parameter recovery method (PRM) for all 150 unthinned stands, (3) the modelling and prediction properties were tested by adopting the 159 independent stands.

For *R* distribution, parameter estimates for *p* (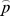), *q* (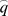), r (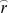) were obtained by employing NRM. For Weibull equation, parameter estimates for *a* (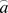), *b* (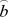) and *c* (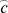) were obtained by employing NRM and MLEM. Because the location parameter *a* of the three-parametric Weibull function was often viewed as the minimum observed diameter or its multiple [32], parameter *a* was defined as the lower limit of the minimum diameter class when MLEM was adopted.

PPM and PRM were used to build stand-level diameter distribution models [5], [35]–[38]. PPM means direct regression of parameters from stand characteristics, and PRM means to recalculate parameters after estimating percentiles or some key point on equation curves from stand characteristics. Some parameters of *R* distribution and Weibull were regressed against stand characteristics which included stand age, site index, planting density, average height, dominant height and quadratic mean DBH (*D_g_*), some parameters were derived from the moments of the diameter distribution, which were themselves estimated from stand characteristics. *D_g_* is calculated by 
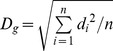
, where 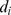 is the diameter values in a stand, *n* is the total number of trees in the stand.

For *R* distribution, when PPM and PRM are simultaneously adopted to predict diameter distributions of stands used for model testing, based on preliminary graphical and correlation analyses, the parameter prediction equations for estimates for 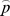, 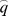 and 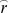 are given by Eqs. (4), (5) and (6), respectively
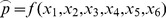
(4)

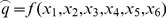
(5)

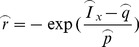
(6)


where 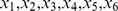 are the stand characteristics, and 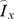 is the estimate of abscissa of inflection point of stand diameter distribution, which can be derived by
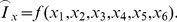
(7)


When only PRM is the adopted method, besides Eq. (6), Eqs. (8) and (9) can be used to recover the parameters
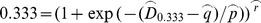
(8)

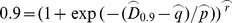
(9)


where 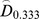 and 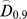 are the diameter at the percentile 0.333 and 0.9 on the distribution curve. Like 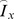, 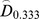 and 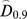 can be predicted by the same formula as Eq. (7).

For the three-parametric Weibull function, the PPM and PRM are simultaneously adopted to predict stand parameters. Parameters 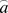 and 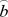 can be estimated as parameters 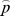 and 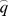 of *R* distribution. Parameter 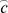 can be solved from the cumulative distribution function of Weibull by Eq. (10).
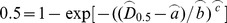
(10)


The reason that the 0.5 percentile was adopted is that this percentile was near the inflection point of the distribution curve [15]. The prediction of 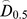 can be achieved by using the same method as 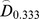 and 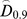 of *R* distribution.

To further test the modelling and prediction properties of *R* distribution and Weibull, another database comprised of 159 diameter frequency distributions was used to test the models.

### Comparison of the Models

The application effect of *R* distribution and three-parametric Weibull function was examined by comparing the residual sum of square (*RSS*) and coefficient of determination (*R^2^*). The residual sum of square and coefficient of determination were respectively calculated as
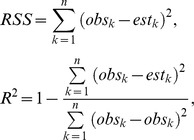



where *obs_k_* and *est_k_* are the observed and predicted diameter frequency for diameter class *k*, and *n* is the number of diameter classes in a sample stand.

We also used the Kolmogorov-Smirnov test to test the goodness of fit of distribution functions. SAS 9.1 and EXCEL 2003 was adopted for parameter estimation and model evaluation.

## Results and Discussion

### Modelling Results of *R* Distribution and Three- Parametric Weibull Function

Table 4 shows the mathematical characteristics of parameters and statistics for *R* distribution and the three-parametric Weibull function derived from the 150 plot measurements. For *R* distribution, the estimates of parameters *p*, *q* and *r* are >0, >0 and <0, respectively, which is identical to the empirical distribution range mentioned in Equation (2). The floating range, mean and standard deviation of parameter *p* or *q* shows that these two parameters are both stable. The total RSS from all stands of *R* distribution (0.1913) was smaller than Weibull distribution (0.2069), and the mean of *R^2^* (*R^2^* = 0.9990) was slightly higher than Weibull distribution (*R^2^* = 0.9988) from every stand while NRM was applied. Although the precision of the two models are both high, *R* distribution presents a more accurate simulation than the three-parametric Weibull function (Table 4). For the three-parametric Weibull function, the precision of NRM is far higher than MLEM in the view of total RSS and *R^2^* (Table 4).

**Table 4:** The mathematical characteristics of parameters and statistics for *R* distribution and three-parametric Weibull derived from the 150 plot measurements.

Equations	Methods^a^	Parameters							*R^2^*			Total *RSS*	
		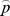	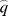	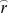	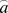	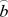	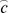	Range		Mean			
*R* distribution	NRM	0.52∼2.57(1.26, 0.44)*	3.39∼18.32(9.40, 3.19)*	−6.83∼–0.30	–	–	–	0.9918∼1		0.9990	0.1913		
Weibull	NRM	–	–	–	0∼12.00	4.53∼17.65	1.93∼8.59	0.9877∼1		0.9988	0.2069		
	MLEM	–	–	–	1.00∼11.00	4.46∼11.53	2.17∼4.83	0.9817∼1		0.9982	0.2814		

a NRM and MLEM refer to nonlinear regression method and maximum likelihood estimates method, respectively. *The values in parenthesis are the mean and standard deviation in sequence.

**Figure 1:**
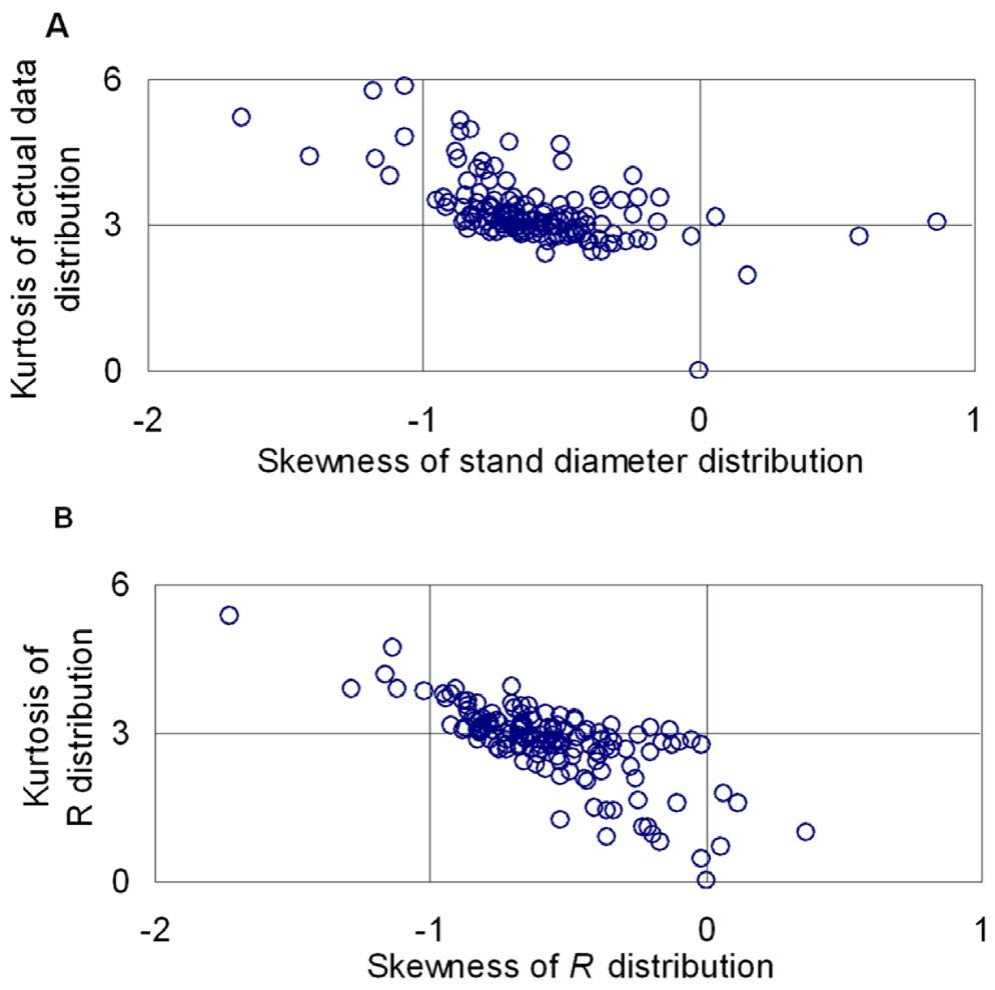
Skewness and Kurtosis values for DBH. **Values for the original data (upper), after the R distribution (lower) are shown.**

The skewness × kurtosis of the original data and *R* distribution is depicted in Figure 1. Each circle dot represents a stand. The distribution range of skewness is similar between the original data and *R* distribution. Most stands have a negative skewness, which is different from inverted J distribution that often happens for the natural stand with positive skewness [2]. The kurtosis obtained from *R* distribution are a little smaller than the original data for some stands, but the value range of kurtosis is same as the actual values. The results mean the shapes modeled by *R* distribution are undistorted and multiple.

**Figure 2:**
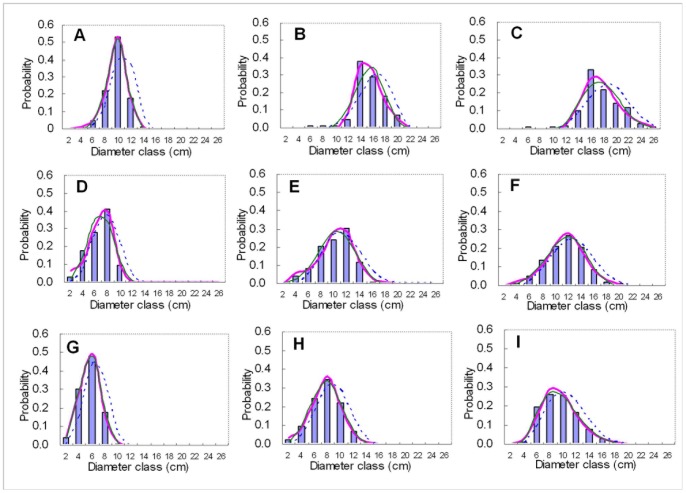
Examples of fitted diameter distributions. The histograms are the observed distribution. The fitted *R* distribution (bold solid line), Weibull distribution obtained from NRM (solid line) and Weibull distribution obtained from MLEM (dashed line). The informations of the nine given stands (A–I) include planting density, stand age and quadratic mean DBH in sequence. (A: 1667 stems/ha, 6 a, 9.8 cm; B: 1667 stems/ha, 12 a, 15.7 cm; C: 1667 stems/ha, 18a, 18.0cm; D: 5000 stems/ha, 6 a, 7.0 cm; E: 5000 stems/ha, 12 a, 10.6 cm; F: 5000 stems/ha, 18a, 12.0cm; G: 10000 stems/ha, 6 a, 5.8 cm; H: 10000 stems/ha, 12 a, 8.2 cm; I: 10000 stems/ha, 18 a, 10.2 cm).

Figure 2 shows both the actual data distribution (histogram), the estimated R distribution shapes (bold solid line), the estimated Weibull distribution obtained from NRM (solid line) and the estimated Weibull distribution obtained from MLEM (dashed line) for a selection of stands from different planting densities, stand ages and quadratic mean DBH. In general *R* distribution and Weibull distribution from NRM both have provided a good fit for all the stands analyzed, and Weibull distribution from MLEM provided a relatively bad fit. In comparison, *R* distribution is more stable than Weibull distribution from NRM, which can be seen from the application of the two distributions to stand 2, 4.

### Theoretical Meaning of Parameters of *R* Distribution

The modelling accuracy and theoretical meanings of parameters are the two most important indexes used to judge whether a function is suitable to modelling diameter distributions and the two indexes are complementary. It is known that *R* distribution has high modelling precision (Table 4). However, do the parameters of *R* distribution have good theoretical interpretation? This question can be answered by discussing the relationship between parameters of *R* distribution and the basic stand characteristics such as stand age, stand density, site index and quadratic mean DBH.

### Theoretical Meaning of Parameters *p* of *R* Distribution

**Figure 3:**
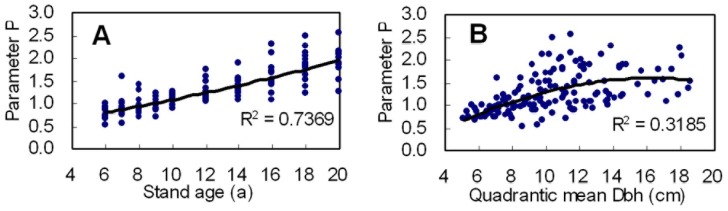
Relationship of parameter p of ***R*** **distribution to stand age (A) and quadratic mean dbh (B).**



Figure 3 shows the relationship of parameter *p* of *R* distribution to stand age and quadratic mean DBH. Parameter *p* increased with increasing stand age and quadratic mean DBH. Liu et al. (2004) [38] also found that the relationship between scale parameter *b* of Weibull distribution and stand age was positive. The relationship of parameter *p* and stand age and quadratic mean DBH were well approximated by the brief second polynomial. The resultant parameter prediction equation for predicting *p* is given by Eq. (11).

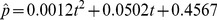
(11)where *t* refers to stand age. The result of analysis of variance showed that Parameter *p* had significant relativity with stand age at the 0.0001 significance level. The coefficients of determination (*R^2^*) of the second polynomial between parameter *p* and stand age, planting density, site index and quadratic mean DBH were respectively 0.7369, 0.0235, 0.0093 and 0.3185, and the positive or negative relativities of them can reasonably interpret the theoretical meaning of parameter *p* as a scale parameter of diameter distribution. According to the definition of scale parameter of Weibull distribution [4], parameter *p* can be viewed as the scale parameter of *R* distribution.

### The Theoretical Meaning of Parameters *q* of *R* Distribution

**Figure 4:**
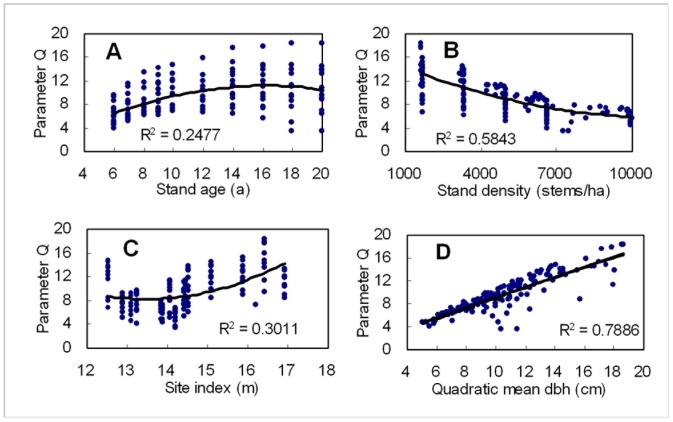
Relationship of parameter q of ***R*** **distribution to stand age (A), stand density (B), site index (C) and quadratic mean dbh (D).**

The relationship of parameter *q* of *R* distribution to stand age, stand density, site index and quadratic mean DBH is illustrated in Figure 4. Parameter *q* increased with increasing stand age, site index and quadratic mean DBH, while it decreased with increasing stand density. The coefficients of determination (*R^2^*) of the second polynomial between parameter *q* and stand age, stand density, site index and quadratic mean DBH were respectively 0.2477, 0.5843, 0.3011 and 0.7886. It is obvious that quadratic mean DBH has the biggest effect on parameter *q* among these stand characteristics. The parameter prediction equation for predicting *q* is given by Eq. (12).
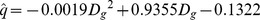
(12)


The coefficients of Eq. (12) were significant at the 0.0001 significance level. From Figure 4, it can be known that the positive or negative relativities of parameter *q* and the four stand characteristics can reasonably interpret the theoretical meaning of parameter *q* as a location parameter of diameter distribution. For Weibull distribution, the location parameter *a* also increased with increasing quadratic mean dbh and stand age [34], [39]. However we should know the fact that the Weibull location parameter must be smaller than observed diameter in a stand. Based on our experience with the data set and other data sets, the convergence and precision of Weibull function are sensitive to the location parameter *a*. Some researchers regarded parameter *a* as minmum diameter in a stand or its times [4,340,41], such as 1/3, 1/2, and 1. And it is difficult to get a common sense. However, for *R* distribution, there is no strictly restrictive condition about location parameter *q* (eq. 2). According to the form of *R* distributin function, parameter *q* can be regarded as the location parameter of *R* distribution, which may make *R* distribution become a new promising diameter distribution.

In previous studies, Duan et al [42] discovered Richards function was suitable for modelling diameter distribution, but did not realize the underlying relationship between parameter *B* and *k* in Richards function. They thought parameter *B* had poor theoretical interpretation. For *R* distribution, parameter *q*, composed of parameters *B* and *k*, obviously has good theoretical meaning, and proved easy to converge. This might lead to the use of *R* distribution as a new diameter distribution.

### Theoretical Meaning of Parameters *r* of *R* Distribution

**Figure 5:**
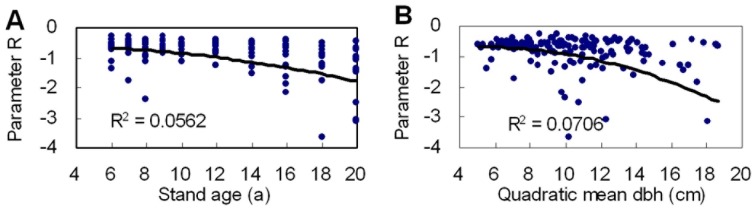
Relationship of parameter r of ***R*** **distribution to stand age (A) and quadratic mean dbh (B).**

Figure 5 shows the relationship of parameter *r* of *R* distribution to stand age and quadratic mean DBH. Parameter *r* decreased with increasing stand age and quadratic mean DBH. The result of analysis of variance showed that parameter *r* had significant relativity with stand age and quadratic mean DBH at the 0.01 significance level. The coefficients of determination (*R^2^*) of the second polynomial between parameter *r* and stand age and quadratic mean DBH were 0.0562 and 0.0706, respectively.

**Figure 6:**
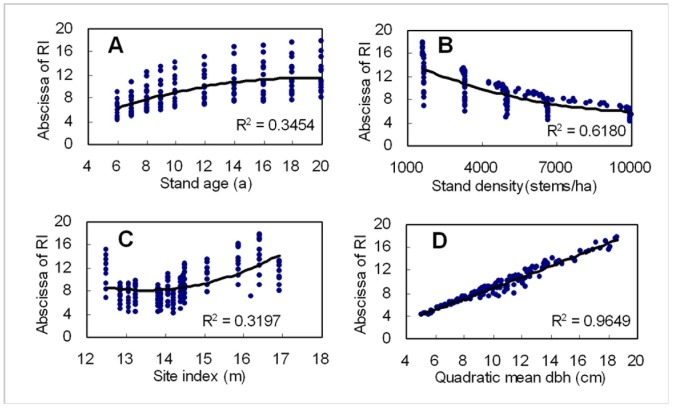
Relationship of abscissa of inflection point of ***R*** **distribution to stand age (A), stand density (B), site index (C), quadratic mean dbh (D) where RI refers to inflection point of R distribution.**



For a sigmoid curve, the location of inflection point decides its shape at some extent. Parameters *p*, *q* and *r* together decide the abscissa of inflection point of *R* distribution (Table 3). As shown in Fig. 6, the abscissa of inflection point of *R* distribution increased with increasing stand age, site index and quadratic mean DBH, and decreased with increasing stand density. The coefficient of determination (*R^2^*) of the second polynomial between abscissa of inflection point and stand age, stand density, site index and quadratic mean DBH were 0.3454, 0.6180, 0.3197 and 0.9649, respectively. The deep relationship between the abscissa of inflection point and quadratic mean DBH can be described by Eq. (13).

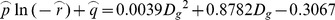
(13)where *D_g_* refers to quadratic mean DBH.

**Figure 7:**
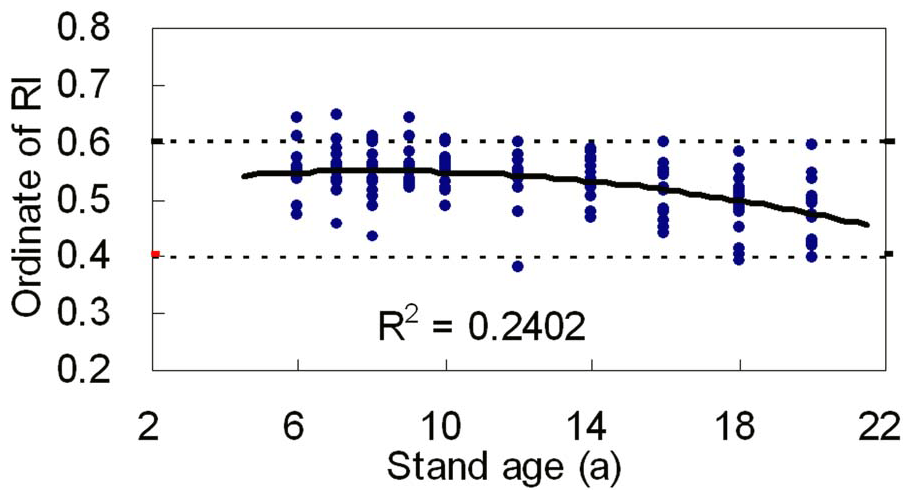
Relationship of ordinate of inflection point of ***R*** **distribution to stand age where RI refers to inflection point of** ***R*** **distribution.**

**Figure 8:**
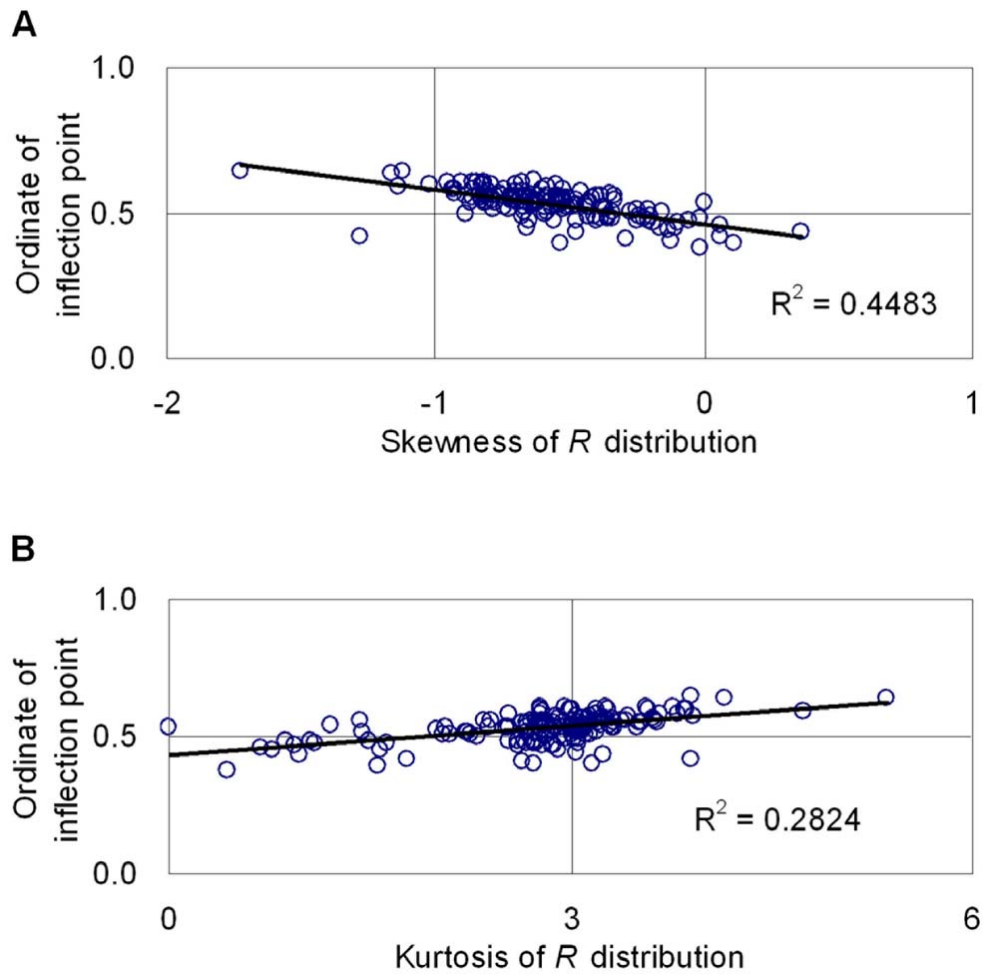
Relationship between the ordinate of inflection point of ***R*** **distribution and its skewness (upper) and kurtosis (lower).**

The value of 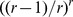, being the ordinate of inflection point of *R* distribution, is decided by parameter *r*. Figure 7 shows the relationship of ordinate of inflection point of *R* distribution to stand age. The ordinate of inflection point decreased with increasing stand age. The coefficient of determination was 0.2402, which means that the ordinate of inflection point of *R* distribution can show the change of distribution shape with age in some extent. Besides, the potential relationship between the ordinate of inflection point of *R* distribution and its skewness is showed in Figure 8. It is found that the ordinate of inflection point of *R* distribution has significant relativity with its skewness and kurtosis, the linear coefficient of determination are 0.4483 and 0.2824 respectively. This phenomenon shows that the size of inflection point of R distribution can report the shape of stands in some extent.

Due to the act of parameter *r* in inflection point of *R* distribution, it can be concluded that parameter *r* has a deep relationship with the shape of *R* distribution. Besides, the significant correlation between abscissa and ordinate of inflection point of *R* distribution and stand characteristics indirectly shows that parameter *r* is flexible and theoretical. Accordingly, parameter *r* may be considered as the shape parameter of *R* distribution.

### Distribution of Inflection Point of *R* Distribution

*R* distribution has a flexible inflection point. The variation range of the ordinates of inflection points is 0.3787∼0.6436, and 91.33 percent of them distribute in the range of 0.4∼0.6. Therefore, we can conclude that the main distribution range of inflection points for the cumulative diameter distribution of stands was 0.4∼0.6.

### Functions for the Estimation of Stand Parameters

Stepwise regression analysis was applied to build the relationship between the parameters of two distributions and the stand characteristics composed of stand age, planting density, site index and so on at a 0.5 risk level. The regressions are on the 150 estimation stands. When nonlinear regression method were adopted, the resultant parameter prediction equations for predicting 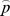, 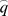, 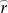, 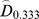, 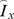, 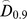, 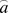, 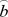, 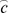 and 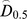 are given by Eqs. (14)–(22), respectively (Table 5). When maximum likelihood estimates method adopted, the resultant parameter prediction equations for predicting 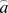, 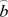 and 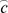 are given by Eqs. (23)–(26), respectively (Table 5).

**Table 5:** The regression expressions and coefficients of determination (*R^2^*) between parameters or diameters at the key points and stand characteristics for *R* distribution and three-parametric Weibull.

Distribution function	Parameter prediction equation	*R^2^*	Number
*R* distribution	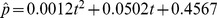	0.7369	(11)
	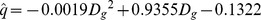	0.7886	(12)
	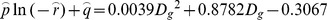	0.9649	(13)
	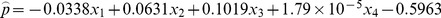	0.7534	(14)
	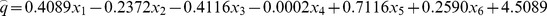	0.8418	(15)
	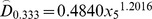	0.9778	(16)
	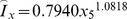	0.9649	(17)
	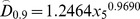	0.9835	(18)
			
Weibull	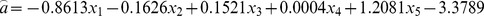	0.5181	(19)
	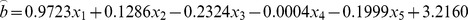	0.5316	(20)
	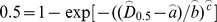	0.9988	(21)
	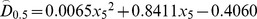	0.9881	(22)
	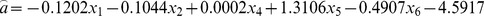	0.8296	(23)
	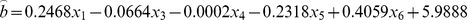	0.5635	(24)
	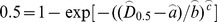	0.9982	(25)
	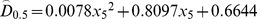	0.9884	(26)

^a^The variables 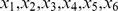 are, respectively, dominant height, stand age, site index, planting density, quadratic mean DBH and mean height, 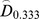 and 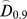 are the diameter estimates at the percentile 0.333 and 0.9 on the *R* distribution curve respectively, 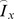 is the estimate of abscissa of inflection point of *R* distribution, 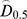 is diameter estimate at the percentile 0.5 on the Weibull curve.

Obviously, because of weak relativity, parameter 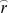 of *R* distribution was not adaptive to be directly predicted by stand characteristics, which could be indirectly obtained through Eqs. (6) or (13). For parameter 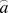, 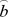 of Weibull distribution, the coefficients of determination of second degree polynomials between the other parameter and above-mentioned stand variable were all smaller than those of Eqs. (19), (20), (21), and (22), respectively. Therefore, the five equations were adopted to predict the unknown distribution parameters.

Note that for the three-parametric Weibull function, regardless of whether the nonlinear regression method or maximum likelihood estimates method is used, 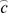 always has significant relativity with planting density and stand age at the 0.0001 significance level. However, under the consideration of relativity, parameter 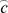 is obtained through Eqs. (21) and (22) or Eqs. (25), and (26). It can be found that these functions have different stand characteristics and exponentia, which are mainly concluded by the analysis of theoretical meaning of parameters and the needs of brief and high precision of models. In practice, while considering parameter prediction method, the parameter prediction models that have both acceptable high precision and theoretical meaning are firstly selected, which often include single stand characteristics, then for parameters with high relativity with some stand characteristics, stepwise regression analysis can be applied. While some or single parameter in distribution model with weak relativity with stand characteristics, parameter recovery method can be adopted. Of course, parameter recovery method can be applied in other conditions. Based on the above-mentioned parameter prediction equations that have a high coefficient of determination, parameters of distribution models can be evaluated by introducing the related stand characteristics into these equations.

### Goodness-of-fit for Estimating Diameter Distribution

Data from 159 evaluation subplots provide an opportunity to analyze and compare the accuracy of the *R* distribution and three-parametric Weibull function. In these calculations, two fitting methods (nonlinear regression method and maximum likelihood estimates method) and two parameter estimation methods (PRM and the combination of PPM and PRM) were used.

**Figure 9:**
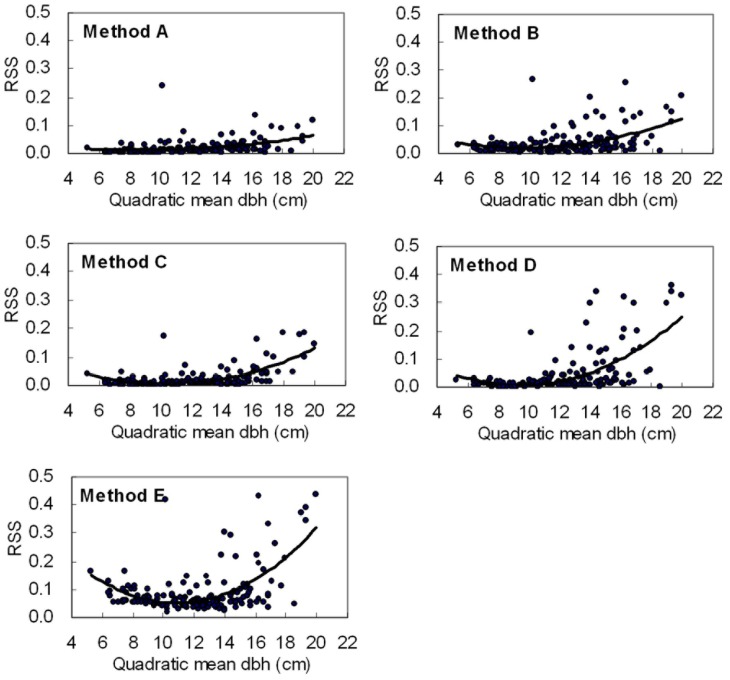
The distribution of the residual sum of square (***RSS*** **) against quadratic mean DBH.** **Method A, Method B, Method C, Method D, Method E are codes in** **Table 6** **.** ****

It was encouraging that *R* distribution was found to have lower *RSS* and higher non-rejection rate than the three-parametric Weibull function (e.g. Table. 6). Figure 9 shows the distribution of the residual sum of square (*RSS*) against quadratic mean DBH, from which we can directly compare the prediction effects of the five methods. Method A has the highest precision among the five methods, which shows *R* distribution can accurately estimate diameter distribution of most future stands using the combination of PPM and PRM under the condition that only two stand characteristics are known (e.g. Table. 6). For method A, the result of the Kolmogorov -Smirnov test showed the null hypothesis that the observed and fitted distributions that are the same cannot be rejected for 128 out of 159 stands (80.50%). However, in view of the practical application of distribution models to stand management, method B, based on *R* distribution, would be a better option because of its dependence on stand characteristics related to stand density and site quality and because its non-rejection rate reached 73.58% for the total test data. Methods B and D used the same parameter recovery method and numbers of stand characteristics and had a nearly equal non-rejection rate, which showed *R* distribution had a distribution function equally as good as three-parametric Weibull function. Furthermore, because the iterated function of *R* distribution was easier to converge than three-parametric Weibull function when using nonlinear regression method to predict parameters, *R* distribution would have a wide application prospect.

For *Chinese fir* plantations, the non-rejection rate of unthinned stands and thinned stands did not have obvious differences when methods A, B, C and D were adopted to predict the diameter distributions of future stands (e.g. Table. 6).

Additionally, for the three-parametric Weibull function, the nonlinear regression method is a more effective approach than the maximum likelihood estimates method in the estimation system of stand diameter distribution (e.g. Table. 6).

## Conclusion

Based on analysis of the disadvantages of the three-parametric Weibull function, this study develops a promising distribution function (*R* distribution), which is a new and essential exploration in the study of parametric methods. We conclude that: (1) *R* distribution has a more accurate simulation than the three-parametric Weibull function while modelling diameter distributions of *Chinese fir* plantations; (2) the parameters *p*, *q* and *r* of *R* distribution proved to be its scale, location and shape parameters, and have a deep relationship with stand characteristics; this means the parameters of *R* distribution have good theoretical interpretation; (3) the main distribution range of inflection points for the cumulative diameter distribution of *Chinese fir* plantations was 0.4∼0.6; (4) the goodness-of-fit test showed the diameter distributions of unknown stands can be accurately estimated by applying *R* distribution and with regards to modelling precision and theoretical interpretation, method B (table 6) may be the most suitable choice due to its good convergence, high precision and including multiple stand characteristics.

**Table 6:** The statistics of different evaluation methods for *R* distribution and the three-parametric Weibull function derived from the 159 stands used for goodness-of-fit tests.

Equations	Codes	Methods^a^		Related equations	Numbers of variables	The sum of *RSS*	Non-rejection rate		
		Modelling	Prediction				Un-thinned stands	Thinned stands	Total
*R* distribution	Method A	NRM	PPM and PRM	(11), (12), (13)	2	3.0920	80.95%	80.21%	80.50%
	Method B	NRM	PPM and PRM	(13), (14), (15)	6	5.7097	68.25%	77.08%	73.58%
	Method C	NRM	PRM	(6), (8), (9), (16), (17), (18)	1	3.5058	82.54%	78.13%	79.87%
									
Weibull	Method D	NRM	PPM and PRM	(19), (20), (21),(22)	6	6.9981	73.02%	71.88%	72.33%
	Method E	MLEM	PPM and PRM	(23), (24), (25), (26)	6	14.2322	6.35%	19.79%	15.09%

a NRM and MLEM refer to nonlinear regression method and maximum likelihood estimates method, respectively. Among the 159 stands, 63 stands came from unthinned stands, 96 stands came from thinned stands.
